# Association Between Maternal Literacy and Child Immunization According to the Expanded Program on Immunization Schedule in a Primary Health Care Center of a Squatter Settlement in Karachi

**DOI:** 10.7759/cureus.43608

**Published:** 2023-08-16

**Authors:** Maryum Anwar, Arshika Faisal, Kainat Jawed, Aamna Yousuf, Imran Shaikh

**Affiliations:** 1 College of Medicine, Ziauddin University, Karachi, PAK; 2 Community Health Sciences, Ziauddin University, Karachi, PAK

**Keywords:** primary healthcare center, children, vaccination, maternal literacy, immunization

## Abstract

Background

An increase in maternal education may influence vaccine administration to a significant extent, therefore reducing the childhood mortality rate. Hence, this survey aims to establish an association between maternal literacy and childhood immunization in children under five years of age.

Methods

A questionnaire-based cross-sectional study was conducted in a primary healthcare center of a squatter settlement in Karachi, Pakistan. Mothers of 250 children under the age of five years were interviewed. We used IBM SPSS Statistics for Windows, version 20 (released 2011; IBM Corp., Armonk, New York, United States) for data analysis to assess the relationship between maternal education and childhood immunization.

Results

The survey revealed that complete vaccination coverage among children under five years of age (n=250) was 71.7%, while 24.6% were partially vaccinated and 2% were unvaccinated. The most common reason for unvaccinated children was the parents' personal choice (80%), while incomplete vaccination was majorly due to a medical condition (30.2%).

Conclusion

According to the survey, maternal educational qualification did not prove to be directly associated with vaccination coverage in children. However, a multi-centered study with larger sample size and multiple populations as targets would provide more accurate outcomes.

## Introduction

Immunization is one of the most effective and cost-efficient public health initiatives [1]. According to the World Health Organization (WHO), 5.2 million children under the age of five die annually. An additional 1.5 million fatalities might be averted, provided that global immunization coverage is improved [2]. For the development of lifelong immunity in a child, complete doses of vaccination under the Expanded Program on Immunization (EPI) are essential. Existing research shows that improved maternal education can have a favorable impact on a family's health outcomes through a balance of power, a better understanding of risks, and the benefits of immunization [3]. Officially, the literacy rate for women, 15 years and older, in Pakistan is around 46.5% [4].

In the last 30 years, the immunization rate of children worldwide has improved from 5% to 80% [1]. Data from different parts of the world show that children of educated mothers are more likely to complete their vaccinations [5]. A study conducted in Africa shows that children of mothers who had completed primary education were about 1.5 times more likely to have received the oral polio vaccine (OPV). Each year of a mother's education results in a 7-9% reduction in under-five mortality [6]. According to the Pakistan Demographic and Health Survey (PDHS), the percentage of children receiving all basic vaccinations has increased by almost 31% from 1990-1991 to 2017-2018 [7]. However, data obtained from slum areas of Karachi, Pakistan, revealed that only 49% of children were fully vaccinated [8].

Although various researchers in Pakistan have looked into several factors influencing childhood immunization, there is a dearth of recent studies concerning the impact of a mother’s literacy level on a child’s vaccination coverage [3]. Maternal education is an extremely vital determinant of childhood immunization and plays an important role in the family’s physical well-being; hence, it is critical to improve literacy among mothers [9]. Therefore, the primary objective of this study is to determine the impact of maternal education on childhood immunization in children under the age of five in a squatter settlement (i.e., a low socioeconomic residential area, which has developed without the legal right to the land or permission from the concerned authorities to build) of Karachi.

## Materials and methods

This study was conducted in the primary healthcare center in Gulshan-e-Sikandarabad, a squatter settlement in Karachi, Pakistan, using a cross-sectional study design. The survey took place over a period of four months, from March 2022 to July 2022. The sample size was estimated using a WHO sample size calculator, where we took the confidence level as 90%, margin of error as 5%, population proportion as 40%, and population size as 10,000. As a result, a total of 250 children were involved. Accordingly, the mothers and guardians of the 250 children under the age of five years with an EPI card, selected via a non-probability convenience sampling technique, were interviewed. Interviewer-based interviews were conducted, using a questionnaire that comprised 11 questions, including the child’s name, age, gender, mother’s educational qualification and occupation, total number of children born to the mother, and their immunization status. The mothers were also questioned regarding the reason for failure to follow up on the child’s subsequent vaccination doses or not getting the child vaccinated at all.

The maternal educational levels were divided into eight categories: no education, madrasa, primary education, secondary education, matriculation or ordinary level (O levels), intermediate or advanced level (A levels), graduate, and postgraduate. Complete immunization in accordance with the EPI schedule was defined as receiving all of the following WHO-recommended basic vaccines: one dose of Bacille Calmette-Guérin (BCG) vaccine, one dose of hepatitis B vaccine, four doses of OPV, three doses of pneumococcal vaccine, two doses of rotavirus vaccine, three doses of pentavalent vaccine, two doses of inactivated polio vaccine (IPV), one dose of typhoid vaccine, and two doses of measles and rubella (MR) vaccines; meanwhile, unvaccinated was defined as not receiving a single dose of any of the vaccines [10]. "Appropriate for age" and "incomplete" were considered as receiving all the vaccinations up to the current age and not receiving the vaccinations according to age or missing doses, respectively. A pilot study was conducted using the same questionnaire prior to the research in which all the questions received satisfactory responses.

Furthermore, all the vaccinated children not accompanied by their parents or guardians, children older than five years, and those without an EPI card, which served as the proof of all the administered vaccines, were excluded from the study. All the interviewees were assured of their confidentiality being maintained throughout the research, prior to the questioning. The interviews were conducted after obtaining verbal consent, and as the data were collected from multiple ethnic groups, the questionnaire was translated to Urdu and Pashto languages for a better understanding of the interviewees.

Ethical approval

Ethical approval was obtained from Ziauddin University Ethics Review Committee, with approval number 0260723MAY4, given before the onset of the study. 

Statistical analysis

IBM SPSS Statistics for Windows, version 20 (released 2011; IBM Corp., Armonk, New York, United States) was used for data analysis, which was purchased and provided to the authors by Ziauddin University. Because our data were categorical, we applied the chi-square test to find the association between the educational qualification of the mothers and the immunization status of their children, and tables and graphs were constructed using Microsoft Excel, version 16.75.2 (Microsoft Corporation, United States).

## Results

**Figure 1:**
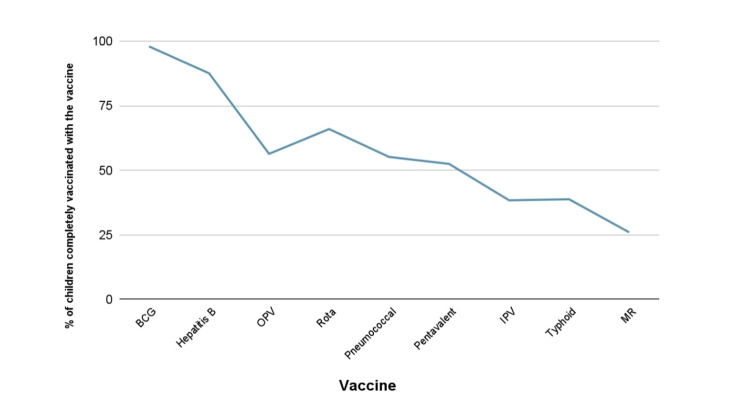
Number of children vaccinated with all the doses of the vaccines. BCG: Bacille Calmette-Guérin, HPV: human papillomavirus, IPV: inactivated polio vaccine, MR: measles and rubella

Out of the 250 total enrolled children, 54% (135) were male while 46% (115) were female. The mean age of the mothers was 26 years, with a range of 17-40 years. The survey revealed that complete vaccination coverage according to the EPI schedule among children under the age of five years (n=250) was 73.2% (183), while 24.8% (64) were partially vaccinated and 2.0% (five) were unvaccinated. The highest level of educational qualification among the mothers was postgraduate with a frequency of 2 (0.8%), with the lowest being uneducated with a frequency of 81 (32.4%). Out of the nine EPI vaccines, BCG had the highest number of doses administered among all the children enrolled in the study, as shown in Figure 1.

As Table 1 shows, a total of 245 (98%) children received the BCG vaccine at birth, out of which 32.2% (79) belonged to uneducated mothers, 19.2% (47) to those with primary education, 24.9% (61) to those who had only attended madrasa, and 23.7% (58) to those with an educational qualification of secondary level or higher. Meanwhile, 2% (five) of the mothers who did not get their children vaccinated either had no education (40%), had been to a madrasa only (20%), or were only educated till the primary level (40%).

**Table 1:** Percentage of children vaccinated with the BCG vaccine and the educational status of their mothers. BCG: Bacille Calmette-Guérin

Educational qualification of mothers	Percentage of children receiving BCG vaccine (%) (n=245)
Uneducated	32.2
Madrasa	24.9
Primary	19.2
Secondary	23.7
Matric/O levels	0
Intermediate/A levels	0
Graduate	0
Postgraduate	0

As shown in Table 2, for the MR vaccine, 26% (65) children were completely vaccinated with two doses and 10.8% (27) had received the first dose appropriate for their age. Meanwhile, 2% (five) had only received the first dose even after the age of 15 months and 6.8% (17) children had not received either of the two doses of the vaccine appropriate for their age. The remaining 54.4% (136) children were under the age of nine months and hence were not eligible for the vaccine. Mothers who did not choose to vaccinate their children with the MR vaccine or failed to follow up for the second dose included those who were uneducated (40%), had attended madrasa only (15%), and studied till the primary (20%) level or secondary level and higher (25%). Table 3 shows the immunization status of children with the MR vaccine for each maternal educational qualification category. The most prevalent reason for an unvaccinated child was the mother’s or family’s personal choice (80%), while incomplete or partial vaccination was primarily due to either a medical condition (30.2%) or negligence and failure to follow up for subsequent doses (14.2%). Out of the 63 partially vaccinated children, 20 (31.7%) failed to receive the hepatitis B vaccine due to its unavailability at the primary healthcare center.

**Table 2:** Immunization status of the children against the MR vaccine. MR: measles and rubella

Immunization status with the MR vaccine	Percentage of children (%) (n=250)
Completely vaccinated (first and second dose)	26
Vaccinated appropriately for age with the first dose	10.8
Incompletely vaccinated	2
Not vaccinated	6.8
Ineligible for vaccine	54.4

**Table 3:** Percentages of educational qualification of mothers against immunization status of children with MR vaccine. MR: measles and rubella

Educational qualification of mothers	Completely vaccinated (%) (n=65)	Vaccinated appropriately for age with the first dose (%) (n=27)	Incompletely vaccinated (%) (n= 5)	Not vaccinated (%) (n= 17)	Ineligible for vaccine (%) (n=136)
Uneducated	24.6	25.9	60	29.4	36
Madrasa	35.4	25.9	0	23.5	21.3
Primary	13.8	25.9	20	23.5	20.5
Secondary	7.7	11.1	0	11.8	10.3
Matric/O levels	15.4	7.4	20	11.8	10.3
Intermediate/A levels	1.5	0	0	0	0
Graduate	0	3.7	0	0	0.7
Postgraduate	1.5	0	0	0	0.7

Table 4 shows the association between maternal educational qualification and the vaccination status of children. As our data were categorical, in order to assess the association between the two variables, we applied a chi-square test of independence. The test showed that the relationship between maternal educational qualification and the vaccination status of children was not significant (p > 0.05). 

**Table 4:** Association between maternal educational qualification and the vaccination status of children. BCG: Bacille Calmette-Guérin, OPV: oral polio vaccine, IPV: inactivated polio vaccine, MR: measles and rubella

Vaccine	Educational qualification of mothers	Vaccination status				Chi-square	P-value
		Complete		Incomplete			
		n	%	n	%		
BCG	No education	81	32.4	0	0	5.209	0.634
	Madrasa	61	24.4	1	0.4		
	Primary	47	18.8	2	0.8		
	Secondary	24	9.6	0	0		
	Matric/O levels	29	11.6	0	0		
	Intermediate/A levels	1	0.4	0	0		
	Graduate	2	0.8	0	0		
	Postgraduate	2	0.8	0	0		
Hepatitis B	No education	71	28.4	10	4	6.018	0.538
	Madrasa	58	23.2	4	1.6		
	Primary	40	16	9	3.6		
	Secondary	19	7.6	5	2		
	Matric/O levels	26	10.4	3	1.2		
	Intermediate/A levels	1	0.4	0	0		
	Graduate	2	0.8	0	0		
	Postgraduate	2	0.8	0	0		
OPV	No education	26	18.4	35	14	6.476	0.485
	Madrasa	38	15.2	24	9.6		
	Primary	28	11.2	21	8.4		
	Secondary	9	3.6	15	6		
	Matric/O levels	16	6.4	13	5.2		
	Intermediate/A levels	1	0.4	0	0		
	Graduate	2	0.8	0	0		
	Postgraduate	1	0.4	1	0.4		
Rotavirus	No education	81	20.8	29	11.6	5.553	0.593
	Madrasa	62	18	17	6.8		
	Primary	49	12.4	18	7.2		
	Secondary	24	5.2	11	4.4		
	Matric/O levels	29	7.6	10	4		
	Intermediate/A levels	1	0.4	0	0		
	Graduate	2	0.8	0	0		
	Postgraduate	2	0.8	0	0		
Pneumococcal	No Education	45	18	36	14.4	6.220	0.514
	Madrasa	37	14.8	25	10		
	Primary	28	11.2	21	8.4		
	Secondary	9	3.6	15	6		
	Matric/O levels	15	6.0	14	5.6		
	Intermediate/A levels	1	0.4	0	0		
	Graduate	2	0.8	0	0		
	Postgraduate	1	0.4	1	0.4		
Pentavalent	No education	45	18	36	14.4	6.220	0.514
	Madrasa	37	14.8	25	10		
	Primary	28	11.2	21	8.4		
	Secondary	9	3.6	15	6		
	Matric/O levels	15	6.0	14	5.6		
	Intermediate/A levels	1	0.4	0	0		
	Graduate	2	0.8	0	0		
	Postgraduate	1	0.4	1	0.4		
IPV	No education	27	10.8	54	21.6	4.336	0.740
	Madrasa	28	11.2	34	13.6		
	Primary	18	7.2	31	12.4		
	Secondary	8	3.2	16	6.4		
	Matric/O levels	12	4.8	17	6.8		
	Intermediate/A levels	1	0.4	0	0		
	Graduate	1	0.4	1	0.4		
	Postgraduate	1	0.4	1	0.4		
Typhoid	No education	27	10.8	54	21.6	5.562	0.592
	Madrasa	29	11.6	33	13.2		
	Primary	17	6.8	32	12.8		
	Secondary	8	3.2	16	6.4		
	Matric/O levels	13	5.2	16	6.4		
	Intermediate/A levels	1	0.4	0	0		
	Graduate	1	0.4	1	0.4		
	Postgraduate	1	0.4	1	0.4		
MR	No education	17	6.8	64	25.6	12.982	0.528
	Madrasa	22	8.8	40	16		
	Primary	9	3.6	40	16		
	Secondary	5	2	19	7.6		
	Matric/O levels	10	4	19	7.6		
	Intermediate/A levels	1	0.4	0	0		
	Graduate	0	0	2	0.8		
	Postgraduate	1	0.4	1	0.4		

## Discussion

A mother’s education has been frequently suggested to be the most crucial factor determining differentials in a child's health outcomes [11]. As stated by Jamil et al. in their article, a mother may be less likely to complete her child's immunization according to the schedule if she has only a limited understanding of vaccination dosages and timings [12].

Numerous advantages of education could explain why educated women are better able to obtain medical attention for their children. Educated mothers, for example, might be more knowledgeable about best medical practices and more aware of the advantages of receiving medical care [11]. Another theory is that a mother with education might be more empowered and have well-paying jobs in developing nations, which would ultimately be good for the child's health [3].

In this study, we observed the relationship between maternal education and child immunizations under the EPI. The findings from our research showed that there was no significant association between the two variables. The majority of children who had received the BCG vaccine accordingly after birth belonged to mothers with either no education (32.2%) or ones who had only attended madrasa (24.8%). Contrary to this, the mothers who did not choose to vaccinate their children with the MR vaccine or failed to follow up for the second dose comprised those uneducated or had studied at a madrasa only (55%). Moreover, our data revealed that vaccination coverage is highest with BCG, the first vaccine administered after birth, and declines steadily with subsequent vaccinations and children’s age, as illustrated in Figure 1. 

The findings of our research were suggestive of no relationship between the maternal literacy rate and childhood immunization, contrary to the results of similar studies previously conducted. A study conducted in rural Nigeria found a particularly strong correlation between full immunization and the mother's knowledge of immunization [5]. According to a research done in Rawalpindi, Pakistan, educated mothers are more likely to fully vaccinate their children than less educated or uneducated mothers [13]. Another similar study conducted in Nigeria, in 2017, showed that complete immunization uptake was higher in children whose mothers were educated and that maternal literacy substantially reduced the magnitude of the effect of maternal education on complete immunization in children [14]. However, some research also revealed a non-significant relationship between the two variables similar to our findings [15], while others showed a negative association [16]. Streatfield et al. observed in a survey done in two villages in Indonesia, although widespread, knowledge regarding immunization was only marginally influenced by a mother’s greater educational qualification. A significant association was also found between knowledge of immunization and immunization levels, even for illiterate mothers [17]. 

Furthermore, the results of our survey revealed that the most prevalent reason for an unvaccinated child, in about 80% of the children, was the family’s personal choice to not immunize their child. Women who are more educated may be able to take on an active, assertive position in their households and in the community, allowing them to demand better medical care for their children, and are known to have better linguistic abilities to use in their dealings with contemporary healthcare environments [11,17].

Our study was conducted at a single primary health center and collected data from different ethnic groups, including immigrants from Khyber Pakhtunkhwa and Afghanistan. A study done in Brent, London, in 2003, which assessed the uptake of the measles, mumps, and rubella 1 (MMR-1) vaccine in six different ethnic groups, revealed that ethnicities may affect immunization to a significant extent because each ethnic group has a unique set of beliefs that influences the choices made by its members [18]. 

Due to the limitations of our study, we were unable to determine the long-term effects of maternal education on childhood immunizations under EPI.

In the long run, increasing women's educational opportunities might benefit maternal and child health in general because educated mothers will make better choices regarding their child's immunization, antenatal care, postnatal care, and possession of a vaccination card [5]. In addition, child immunization rates can be increased by combining other healthcare services with those for mothers and children. These would include educating expectant mothers about the value of vaccinations, providing follow-up systems and services, and using electronic record-keeping and information sharing to avoid duplication or making errors in choice [6].

Our study, however, was center-based and did not take into account populations of different ethnicities, socioeconomic statuses, and religions. Hence, multi-centered research with a larger sample size, targeting multiple populations, would produce more reliable results.

## Conclusions

The level of literacy of mothers did not prove to be directly associated with vaccination coverage for their children. In conclusion, a mother’s advanced educational qualification is not the sole factor positively influencing her child’s immunization status. Other maternal factors, regardless of the level of literacy, might play a critical role in the health of children.
